# Specific depletion of resident microglia in the early stage of stroke reduces cerebral ischemic damage

**DOI:** 10.1186/s12974-021-02127-w

**Published:** 2021-03-23

**Authors:** Ting Li, Jin Zhao, Wenguang Xie, Wanru Yuan, Jing Guo, Shengru Pang, Wen-Biao Gan, Diego Gómez-Nicola, Shengxiang Zhang

**Affiliations:** 1 Gansu Key Laboratory of Biomonitoring and Bioremediation for Environmental Pollution, School of Life Sciences, Lanzhou University, No. 222 South Tianshui Road, Lanzhou, Gansu 730000 People’s Republic of China; 2 Molecular Neurobiology Program, The Kimmel Center for Biology and Medicine of the Skirball Institute, Department of Neuroscience and Physiology, New York University School of Medicine, New York, NY 10016 USA; 3 Centre for Biological Sciences, University of Southampton, South Lab and Path Block, Mail Point 840 LD80C, Southampton General Hospital, Tremona Road, Southampton, SO16 6YD UK

**Keywords:** Microglia, Depletion, Ischemia, Inflammation, Function

## Abstract

**Background:**

Ischemia can induce rapid activation of microglia in the brain. As key immunocompetent cells, reactive microglia play an important role in pathological development of ischemic stroke. However, the role of activated microglia during the development of ischemia remains controversial. Thus, we aimed to investigate the function of reactive microglia in the early stage of ischemic stroke.

**Methods:**

A Rose Bengal photothrombosis model was applied to induce targeted ischemic stroke in mice. CX3CR1^CreER^:R26^iDTR^ mice were used to specifically deplete resident microglia through intragastric administration of tamoxifen (Ta) and intraperitoneal injection of diphtheria toxin (DT). At day 3 after ischemic stroke, behavioral tests were performed. After that, mouse brains were collected for further histological analysis and detection of mRNA expression of inflammatory factors.

**Results:**

The results showed that specific depletion of microglia resulted in a significant decrease in ischemic infarct volume and improved performance in motor ability 3 days after stroke. Microglial depletion caused a remarkable reduction in the densities of degenerating neurons and inducible nitric oxide synthase positive (iNOS^+^) cells. Importantly, depleting microglia induced a significant increase in the mRNA expression level of anti-inflammatory factors TGF-β1, Arg1, IL-10, IL-4, and Ym1 as well as a significant decline of pro-inflammatory factors TNF-α, iNOS, and IL-1β 3 days after stroke.

**Conclusions:**

These results suggest that activated microglia is an important modulator of the brain’s inflammatory response in stroke, contributing to neurological deficit and infarct expansion. Modulation of the inflammatory response through the elimination of microglia at a precise time point may be a promising therapeutic approach for the treatment of cerebral ischemia.

**Supplementary Information:**

The online version contains supplementary material available at 10.1186/s12974-021-02127-w.

## Background

Cerebral ischemic stroke is caused by occlusion or narrowing of cerebral arteries which lead to insufficient blood and oxygen supply. Currently, only a minority of patients are eligible for thrombolysis treatment or decompressive surgery, which is known as the most effective clinical means for acute reperfusion and decreasing life-threatening edema, respectively [1–3]. For most patients, effective strategies to rescue or protect injured neurons are not available due to the complicated pathological changes in ischemic tissue. Various cerebral resident and infiltrating cells contribute to the complex pathological events in the infarct tissue. As the main immunological cells of the central nervous system (CNS), microglia are activated and sensitive to ischemic insults and play a key role in the development of ischemic pathology [4, 5].

Microglia are regarded as one of the major players during pathological progression of neurodegenerative diseases and ischemic stroke [6]. Our previous work has shown an intense and continuous microgliosis response in the subacute phase of ischemic stroke, which is mainly derived from local expansion of resident microglia [7]. However, there is still an extensive debate about whether microglia are beneficial or detrimental to tissue repair or functional recovery, especially in ischemic stroke [8–15]. With regards to the function of the activated microglia, some studies propose that reactive microglia enhance their release of superoxide, matrix metalloproteinases, and some cytokines to act as neurotoxic elements after injuries [16]. Activated microglia can directly phagocytose endothelial cells and further potentiate damage to blood brain barrier constituents and cause secondary hemorrhage after ischemia, leading to a worse injury [14, 17]. In addition, activated microglia are also considered as the primary executors of the inflammatory response and participate in the neurogenesis progress and neuronal loss [18–20]. However, other studies have shown that activated microglia exert neuroprotection under brain ischemic conditions and contribute to post-stroke recovery, via production of various anti-inflammatory cytokines and growth factors to promote the restoration of injured brain [4]. Administration of exogenous microglia increases the expression of neurotrophin and protects against neuronal injury in vivo [21]. Some reports have shown that microglia serve as vital scavengers of cellular debris, participating in restoration of tissue homeostasis after ischemic stroke [22, 23]. Furthermore, microglia with pro-neurogenic phenotype are engaged in neurogenesis, which may be important for the restoration of damaged brain after stroke [24]. In general, although reactive microglia are acknowledged to affect ischemic damage, the exact role of microglia remains unclear.

In this study, we took advantage of the CX3CR1^CreER^:R26^iDTR^ mice to investigate the role of reactive microglia during ischemic injury. Based on our previous work which indicated that reactive microgliosis increased continuously in the early stage after ischemia, we selectively depleted microglia in the first 3 days after stroke. Our results demonstrated that depletion of resident microglia in the early stage of ischemic stroke led to a decrease in infarct volume and degenerative neurons, and an improved performance in motor ability. The reduction in ischemic damage after depleting microglia was accompanied with a decrease in the density of iNOS^+^ cells and a significant decline in mRNA expression of several key pro-inflammatory cytokines, as well as a markedly increase in mRNA expression of several anti-inflammatory cytokines. Altogether, our data suggest that selective depletion of resident microglia at an early stage after ischemic stroke relieves cerebral injury and that regulating microglia-mediated inflammatory response may be used as a strategy to treat ischemic cerebrovascular disease.

## Methods

### Mice and microglia specific depletion system

CX3CR1^CreER^:R26^iDTR^ mice were used to specifically delete microglia from the CNS, with Ta and DT administration [25]. The CX3CR1^CreER^:R26^iDTR^ mice were generated based on the same insertion site as the CX3CR1^GFP^ transgenic mice [25], where brain microglia, peripheral monocytes, and a subset of NK cells were fluorescence labeled [26]. After genotype identification, only heterozygous mice containing both *CreER-IRES-EYFP* and *Rosa26-stop-DTR* were used in subsequent experiments. The primer sequences used were listed in Table 1. Mice aged 12-14 weeks (23 ± 3 g) were chosen for this study. To deplete microglia from the brain, Ta (0.4 g/kg mouse, Sigma, Cat# T5648) was firstly given by intragastric administration twice over 3 days. In order to preserve monocytes and NK cells in blood circulation, we waited 10 days for them to renew [25]. After that, DT (0.04 mg/kg mouse, Sigma, Cat# D0564) was intraperitoneally injected for three consecutive days to deplete only microglia but not newborn peripheral cells. All animals were bred at the animal core facility of Lanzhou University, under a 12 h light/12 h dark cycle at 22 ± 2 °C, with clean water and rodent chow ad libitum.

**Table 1:** Primer sequences used to validate CX3CR1^CreER/+^:R26^iDTR/+^ transgenic mice

Gene name	Primer
*Cre ert-common*	5′-AAGACTCACGTGGACCTGCT-3′
*Cre ert-WT*	5′-AGGATGTTGACTTCCGAGTTG-3′
*Cre ert-mutant*	5′-CGGTTATTCAACTTGCACCA-3′
*DTR-common*	5′-AAAGTCGCTCTGAGTTGTTAT-3′
*DTR-WT*	5′-GGAGCGGGAGAAATGGATATG-3′
*DTR-mutant*	5′-GCGAAGAGTTTGTCCTCAACC-3′

### Fluorescence-activated cell sorting analysis

Before sacrificed, 100 μl blood of each mouse was collected from the orbital vein. Then mice were transcardially perfused with 0.01M phosphate-buffered saline (PBS) for dissection of spleen. Spleen were homogenized in PBS and filtered through 70 μm cell strainers. After erythrolysis and centrifugation, leukocytes from the blood samples and homogenized spleens were collected respectively and then stained with anti-CD11b (PE, marker of monocytes and a subset of NK cells, 1:35, Biolegend, Cat# 101207) antibody. The samples were assayed by a BD FACSverse flow cytometer (BD, LSRFortessaTM) to measure the percentage of CD11b-positive cells. FACS data were analyzed by the FlowJo software.

### Ischemic stroke model

Ischemic stroke surgery was conducted 10 min after the first DT administration. A modified photothrombosis model was applied to induce acute ischemic stroke as described previously [7]. In brief, a cranial window ~50 μm in thickness was thinned over the right somatosensory cortex on the ketamine-xylazine anesthetized mouse (20 mg/ml ketamine, 2 mg/ml xylazine, 0.1 ml/20 g mouse), following intravenous injection of Rose Bengal (0.03 mg/g mouse, Sigma, Cat# R3877). The thinned cranial window was then exposed to a beam of exciting light (530 ± 20 nm) in a ~0.4 mm^2^ area for 2.5 min to activate the photoactive dye Rose Bengal. Singlet oxygen generated from irradiated Rose Bengal causes focal endothelial damage, platelet activation and aggregation. These reactions result in embolization of blood vessels within the illuminated region to form acute ischemic stroke. Three days after stroke, the mice were sacrificed for subsequent experiments.

### Histological analysis

After anesthesia with an overdose of urethane, mice were transcardially perfused with PBS followed by 4% paraformaldehyde. Mouse brains were harvested and fixed in 4% paraformaldehyde for 48 h at 4 °C and then sectioned into 30 μm slices by a vibrating microtome (Leica, VT1000S). Sections were stained with Iba1 (microglia marker, 1:500, Wako, Cat# 019-19741), iNOS (1:300, BD, Cat# 610328) or Arg1 (1:300, Boster, Cat# BA3796-2) primary antibodies followed by fluorescently labeled secondary antibodies, and then imaged under an epifluorescence (Olympus, BX51) or a confocal microscope (Olympus, FV1000). For cell density analysis, cells in randomly selected areas (200 μm × 100 μm) in the periphery of damaged zone were counted.

To assess the infarct volume, every fourth brain slice was collected for Nissl staining and then photographed. The infarct area was identified by light staining of cresyl violet (the normal tissue was darkly stained). For the calculation of ischemic infarct volume, the overestimation of infarct area due to edema in ischemic zone was corrected referring to the method of Lohil et al. [27]. The area of the total left hemisphere (non-ischemic, TLH) and the non-infarct region of the right hemisphere (NRH) of each slice were measured by the ImageJ software. The infarcted area of the right hemisphere (IRH) of each slice was calculated as follow: IRH = TLH−NRH. The total infarct volume of brain was calculated by multiplying the sum of infarct area in each slice by the sampling interval distance (120 μm).

To evaluate neurodegeneration, 3-4 coronal slices of the injured region per mice were randomly selected and stained with Fluoro-Jade C (FJC, Sigma, Cat# AG325), which is a high affinity fluorescent marker for degenerating neurons [28]. A modification of staining procedure was performed as follows: in brief, slices were immersed for 3 min in 100% ethanol, 1 min in 70% ethanol, and rinsed in distilled water. Slices were then incubated in 0.06% potassium permanganate solution for 20 min followed by rinsing in distilled water. Slices were then placed into a 0.0001% solution of Fluoro-Jade C (Merck, Cat# AG325) dissolved in 0.1% acetic acid vehicle for 25 min to stain the degenerative neurons. The slices were rinsed, air dried at 37 °C for at least 30 min, cleared in xylene, and then cover-slipped with DPX. For density analysis of FJC-positive cells, randomly selected rectangles (200 μm × 100 μm) were picked on the borders of the infarct area, starting from the interface between FJC-positive and FJC-negative tissue. The number of FJC-positive cells and the area were measured in each rectangle using the ImageJ software.

### Quantitative real-time polymerase chain reaction

Anaesthetized mice were sacrificed at scheduled time and transcardially perfused with PBS, and then the mouse brains were separated and frozen in liquid nitrogen for 30 s. The injured area of brain was dissected out immediately and stored at −80 °C. To evaluate the mRNA expression level of immunomodulatory molecules after ischemia in the presence and absence of resident microglia, brain tissues were ground in liquid nitrogen. RNA extraction, reverse transcription, and qRT-PCR were performed by a modified procedure as described previously [29, 30]. Briefly, total RNA was extracted from homogenate brain tissue using the RNAprep pure Tissue Kit (TIANGEN, DP431) and reverse-transcribed into cDNA using the PrimeScript™ RT reagent Kit with gDNA Eraser (TAKARA, Cat# RR047A) following manufacturer’s protocols. qRT-PCR was performed using a commercial mix (SYBR@ Premix Ex Taq^TM^ II, TAKARA, Cat# RR820A) and a CFX96 Real-Time PCR Detection system (Bio-RAD). The volume of qRT-PCR was 10 μl, comprised of 0.5 μl of each primer (10μmol/l), 1 μl of cDNA, 3μl of ddH_2_O, and 5 μl of SYBR Mix. The PCR amplification process was as follows: denaturation at 95 °C for 30 s, 40 PCR cycles of 95 °C for 5 s, 60 °C for 30 s. Then, a melting step was performed consisting of 5 s at 60 °C and slow heating at a rate of 0.5 °C/s to 95° C with continuous fluorescence measurement. Quantification was performed using the comparative CT method (1000/2^ΔCT^, ΔCT=CT_target gene_−CT_GADPH_). Corresponding primers were self-designed and sequences were shown in Table 2.

**Table 2:** Primer sequences of inflammatory factors

Gene	Forward	Reverse
*GAPDH*	5′-TGAACGGGAAGCTCACTGG-3′	5′-TCCACCACCCTGTTGCTGTA-3′
*TGFβ1*	5′-TGTACGGCAGTGGCTGAACC-3′	5′-CGTTTGGGGCTGATCCCGTT-3′
*Arg1*	5′-TCACCTGAGCTTTGATGTCG-3′	5′-CTGAAAGGAGCCCTGTCTTG-3′
*IL-10*	5′-TGCCTTCAGTCAAGTGAAGACT-3′	5′-AAACTCATTCATGGCCTTGTA-3′
*IL-4*	5′-CAAACGTCCTCACAGCAACG-3′	5′-AGGCATCGAAAAGCCCGA-3′
*Ym1*	5′-ATGGAAGTTTGGACCTGCCC-3′	5′-AGTAGCAGCCTTGGAATGTCTT-3′
*TNF-α*	5′-ATGGCCTCCCTCTCAGTTC-3′	5′-TTGGTGGTTTGCTACGACGTG-3′
*iNOS*	5′-CCCTTCAATGGTTGGTACATGG-3′	5′-ACATTGATCTCCGTGACAGCC-3′
*IL-1b*	5′-GAAATGCCACCTTTTGACAGTG-3′	5′-TGGATGCTCTCATCAGGACAG-3′
*MCP1*	5′-ACGCTTCTGGGCCTGTTGTT-3′	5′-CCTGCTGCTGGTGATTCTCT-3′

### Behavioral tests

The grip strength test was utilized to evaluate the muscle strength. The forelimb grip strength was tested by a modified device which is composed of a grasping triangle frame and a sensitive force transducer (Xinhang, China, JZ300). Mouse tail was dragged backwards with a constant force when its forelimbs gripped the metal bar of a triangular frame. The maximal power of forelimb grip strength was recorded by the Biological Data Acquisition & Analysis System (Taimeng BL-420F, China) when the mouse loosened its forelimbs. Grip strength of each mouse was measured at the scheduled time and the mean value of five replicates was taken for statistics.

The rotarod test was implemented to assess motor coordination and balance of mice. Before surgery and drug treatment, all mice were subjected to pre-training on a rotarod treadmill (Taimeng ZB-200, China) apparatus for 3 days. The mice which were able to walk on the rotarod (accelerated from 10 rpm to 30 rpm within 5 min) for at least 300 s were chosen for subsequent test. For the test, the time of walking on rotarod was recorded three times for each mouse and the mean was used for statistical analysis.

To measure the level of locomotor activity and exploratory behavior of mice, spontaneous activity test was carried out in an apparatus (JLBehv-LAM-4, Shanghai) consisted of a soundproof box, a shuttle-box (25 cm × 25 cm × 30 cm), and an infrared camera mounted on the ceiling. Each mouse was placed in the center of the shuttle-box. After 5 min of adaption in the test chamber, the total distance mice moved was recorded automatically for 30 min by the DigBehv 2.0 software.

Behavioral tests data were obtained on the day the brains were harvested for each mouse. For sham groups, behavioral performance and body weight were recorded at the day after vehicle or DT treatment without photothrombosis surgery. For results of stroke groups, behaviors and weight were recorded on the third day after stroke.

### Statistical analysis

GraphPad Prism software (Version 8.0.2) was used for statistical analysis as described in a previous study [31]. All measurements and evaluations were conducted in a double-blind manner. Effect of Ta and DT was compared using one-way ANOVA. Behavioral tests and qRT-PCR data were compared using two-way ANOVA. In all case where one-, two-way ANOVA was used, Tukey’s test was performed for multiple comparisons [32]. Single comparisons of data were made using two-tailed *t* test. All data are represented as mean ± sem. A *p* value < 0.05 is considered statistically significant and *p* < 0.01 is extremely significant.

## Results

### The CX3CR1^CreER:iDTR^ system efficiently depletes cerebral microglia

**Fig. 1:**
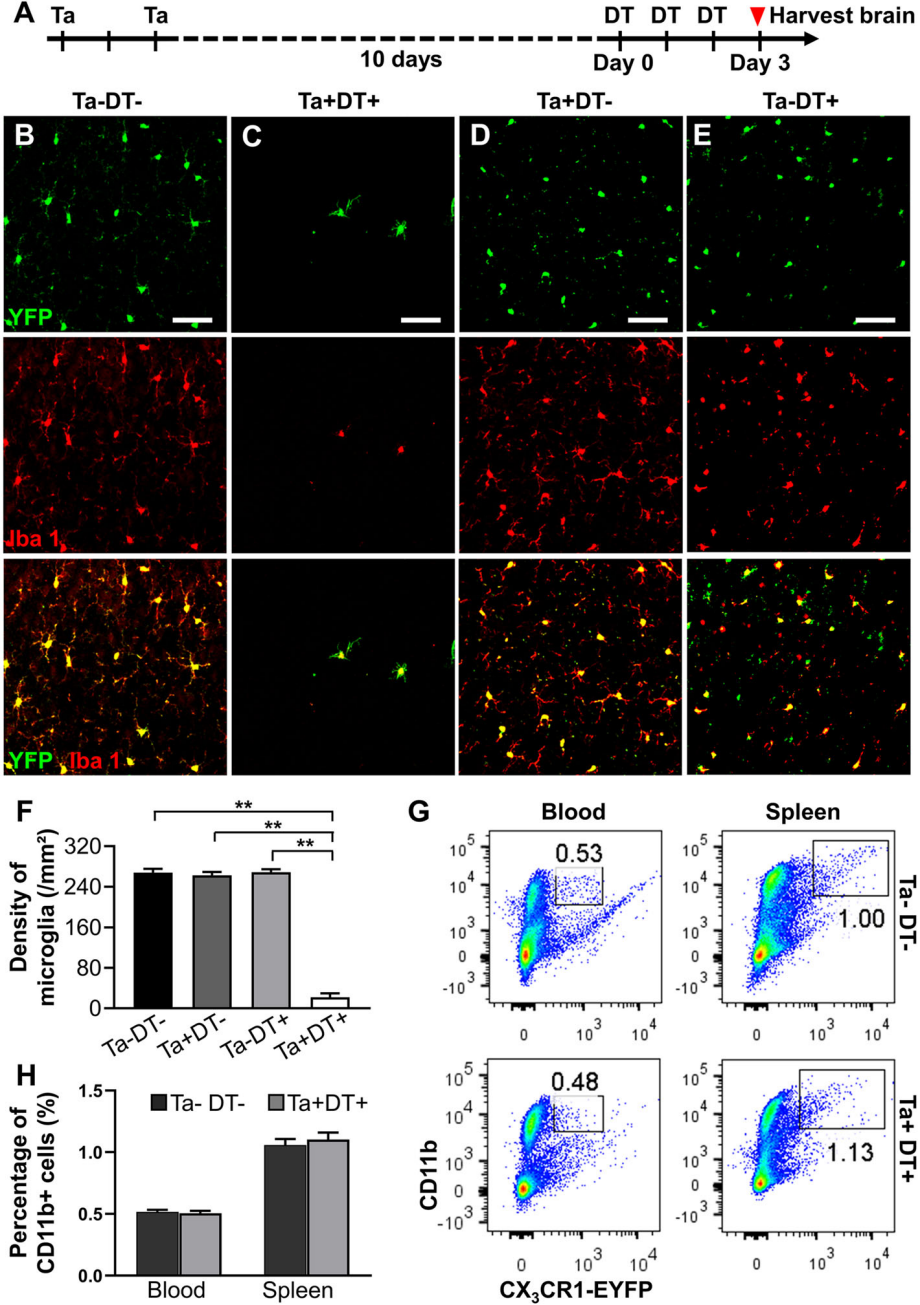
Microglia depletion efficiency of CX3CR1^CreER/+^:R26^iDTR/+^ transgenic mice. (**a**) Timeline of Ta and DT administration in the selective microglial depletion model. (**b**) Represent images of microglia after vehicle treatment. Microglia evenly distributed throughout the cerebral cortex in mice brain without microglial depletion. (**c**) Represent images of microglia after Ta and DT treatment. After drug treatment, most native microglia were depleted and the remnants exhibited appearance similar to activated state and distributed randomly. (**d**) Represent images of microglia only with intragastric administration of Ta but not DT. (**e**) Represent images of microglia only with intraperitoneal injection of DT but not Ta. No significant effect was found on the phenotype and density of microglia in cortex between single- and no-drug groups (**b**-**e** scale bar = 50 μm). (**f**) Densities of microglia in the cerebral cortex with drug treatment or not (*n* ≥5, **p < 0.01). (**g**) FACS analysis showing percentage of CD11b^+^ cells in the blood and spleen of mice after vehicle or Ta^+^DT^+^ treatment. (**h**) Quantification of the FACS analysis results shown in (**g**) (*n* = 4)

In order to evaluate the efficiency of microglia ablation in CX3CR1^CreER/+^:R26^iDTR/+^ mice, we harvested and examined brain slices after Ta and DT treatment in mice without stroke injury (Fig. 1a). In sham^Ta−DT−^ animals, 97.68 ± 1.36% of the yellow fluorescent protein (YFP) expressing microglia (shown in green in Fig. 1) were Iba-1 positive (Fig. 1b). After administration of Ta and DT, only a small number of microglia were found to be scattered in the cortex. The density of resident microglia had a 91.79% reduction compared to vehicle-treated group (sham^Ta−DT−^: 267.85 ± 7.29/mm^2^; sham^Ta+DT+^: 22.00 ± 7.62/mm^2^) (Fig. 1c, f). In addition, we also analyzed the brains of CX3CR1^CreER/+^:R26^iDTR/+^ mice subjected to only Ta or DT and found no significant difference in the microglial density among sham^Ta+DT−^, sham^Ta−DT+^, and sham^Ta−DT−^ groups (Fig. 1b, d-f). Based on the above results, we confirmed that only the combination of Ta and DT could effectively deplete microglia in the brain. Neither Ta nor DT alone affects microglial density under this experimental procedure. To specifically deplete microglia but not CX3CR1^+^ cells in blood circulation, we injected DT at 10 days after Ta to allow recombined cells replaced by non-recombined cells from progenitors. Similar to previous report [25], we observed no significant difference in the percentage of CD11b^+^ cells in the blood or spleen between sham^Ta−DT−^ and sham^Ta+DT+^ mice (Fig. 1g, h). Thus, only microglia but not peripheral CX3CR1^+^ cells in the brain were robustly and specifically depleted after Ta and DT treatment in CX3CR1^CreER/+^:R26^iDTR/+^ mice.

**Fig. 2:**
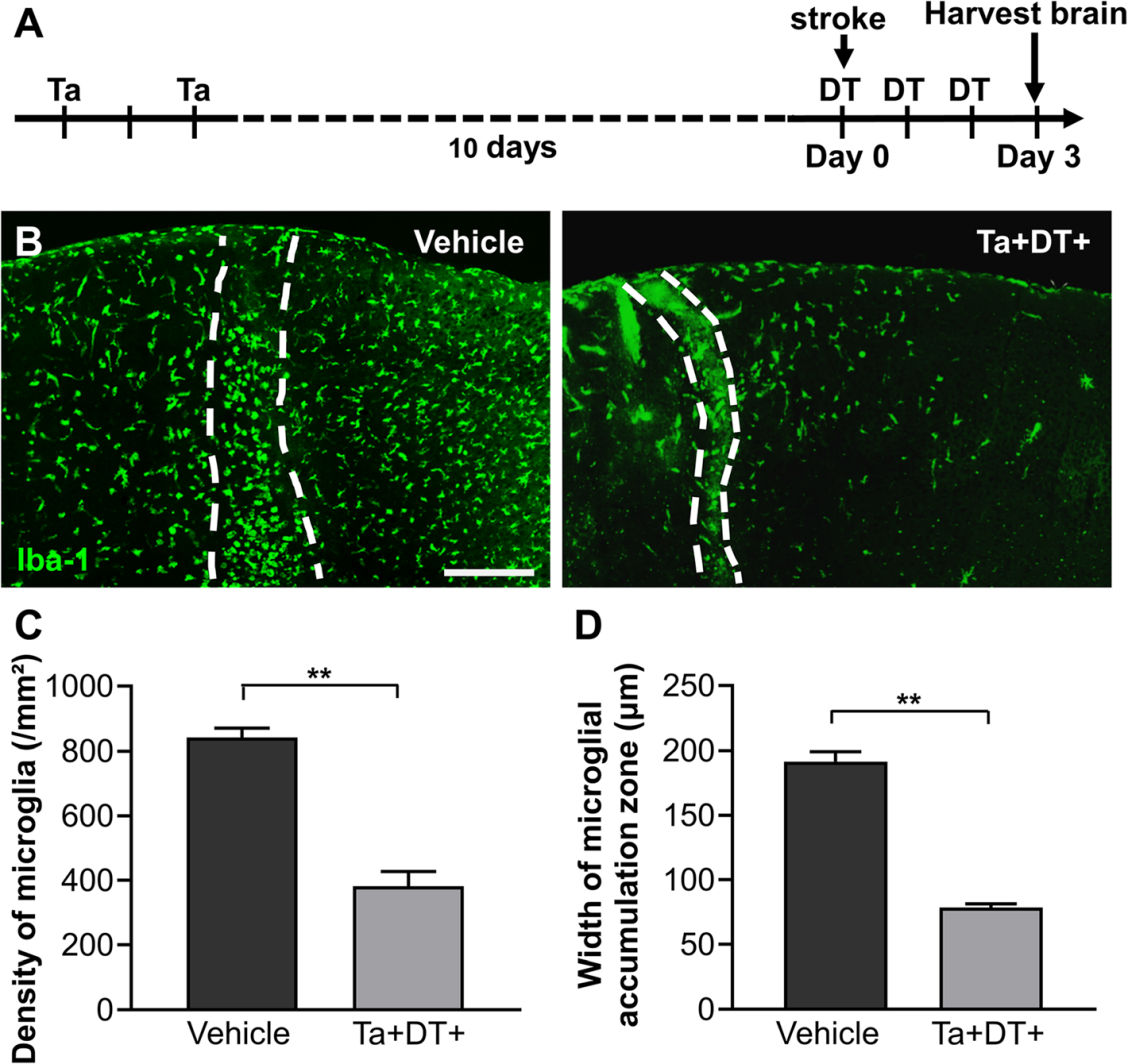
Microgliosis in CX3CR1^CreER/+^:R26^iDTR/+^ transgenic mice 3 days after ischemic stroke. (**a**) Timeline of drug administration and tissue processing 3 days post stroke. (**b**) Distribution of microglia at the lesion site in vehicle-treated mice and microglia-depleted mice 3 days post stroke. Iba-1 was used to highlight microglia in CX3CR1^CreER/+^:R26^iDTR/+^ mice. Microglial accumulation zones were delineated by the dashed lines (scale bar = 200 μm). (**c**) Density of microglia in the accumulation zone with and without microglia depletion. (**d**) Width of the accumulation zone of reactive microglia with and without microglial depletion (*n* ≥ 3, **p < 0.01)

Ischemic stroke has been shown to induce microgliosis with a characteristic of rapid and prolonged accumulation of reactive microglia at the lesion site [7, 33]. In order to explore the role of reactive microglia in the early stage of ischemia, we induced photothrombotic stroke in CX3CR1^CreER/+^:R26^iDTR/+^ mice and depleted microglia by treatment with Ta and DT (Fig. 2a). Three days after stroke, brain slices of stroke^Ta+DT+^ mice were collected to compare with stroke^Ta−DT−^ mice. Compared with vehicle-treated animals, we found that most microglia were depleted after Ta and DT treatment in stroke^Ta+DT+^ mice (Fig. 2b). The residual microglia were found unevenly distributed in the brain (Fig. 2b). Furthermore, microglial depletion made a significant effect on the accumulation zone surrounding the ischemic core (Fig. 2b-d). Compared with the vehicle-treated mice, the density of microglia decreased sharply (841.15 ± 28.97/mm^2^ for stroke^Ta−DT−^ mice versus 381.34 ± 45.75/mm^2^ for stroke^Ta+DT+^ mice) and the width of microglial accumulation zone lessened (191.49 ± 7.60 μm for stroke^Ta−DT−^ mice versus 78.34 ± 2.97 μm for stroke^Ta+DT+^ mice) when resident microglia were depleted in the first 3 days after stroke (Fig. 2c, d). These data supported a significant decrease of activated microglia following depletion after ischemia.

### Microglial depletion reduced infarct volume and degenerating neurons

**Fig. 3:**
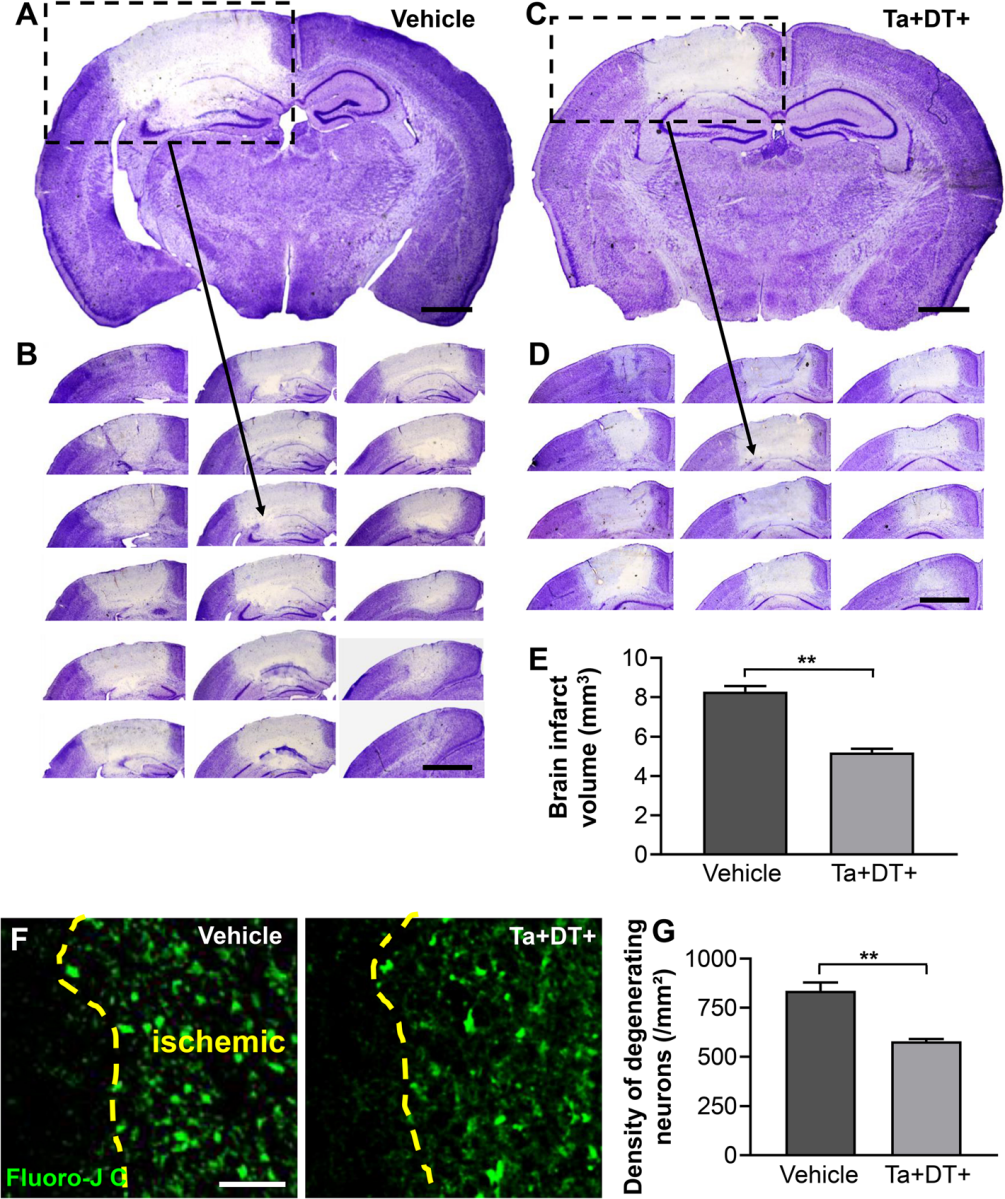
Depletion of microglia decreased the infarct volume and neuronal degeneration 3 days after ischemic stroke. (**a**) Representative Nissl stained coronal brain slice of vehicle-treated mouse after stroke (scale bar = 1 mm). (**b**) Coronal sections in accordance to the boxed region in (**a**) illustrating the whole infarct (scale bar = 2 mm). (**c**) Representative Nissl stained coronal brain slice of microglia-depleted mouse after stroke (scale bar = 1 mm). (**d**) Coronal sections in accordance to the boxed region in (**c**) illustrating the whole infarct (scale bar = 2 mm). (**e**) Calculated brain infarct volumes 3 days post stroke, in mice with Ta and DT treatment or not (*n* ≥ 6, **p < 0.01). (**f**) Representative confocal images of FJC-labeled degenerating neurons in the ischemic areas of stroke^Ta−DT−^ and stroke^Ta+DT+^ mice. The boundaries between ischemic area and normal tissue were delineated by the dashed lines (scale bar = 50 μm). (**g**) Densities of degenerating neurons in the border area underwent microglia depletion or not. Note that neurodegeneration greatly decreased in microglia-devoid mice 3 days after stroke (*n* ≥ 3, **p < 0.01)

To evaluate the effect of microglial depletion on the ischemic damage, we used Nissl staining to assess the infarct area and Fluoro-Jade C staining to quantitate the degenerative neurons after stroke. Our data showed that the infarct volume is 8.28 ± 0.29 mm^3^ 3 days post stroke without drug treatment (Fig. 3a, b, e). We then measured the infarct volume in brains subjected to Ta and DT and found the infarct size was significantly reduced to 5.19 ± 0.19 mm^3^ (Fig. 3c-e). Moreover, we evaluated the density of degenerating neurons in ischemic animals, finding a marked reduction in the density of degenerating neurons 3 days post stroke when microglia were depleted (from 836.31 ± 42.76/mm^2^ in stroke^Ta−DT−^ mice to 578.41 ± 12.07/mm^2^ in stroke^Ta+DT+^ mice) (Fig. 3f, g). These results indicated that microglia ablation was beneficial for delaying the infarct area diffusion and effectively prevented neuronal loss in the early stage post stroke.

### Depletion of resident microglia benefited behavioral recovery

**Fig. 4:**
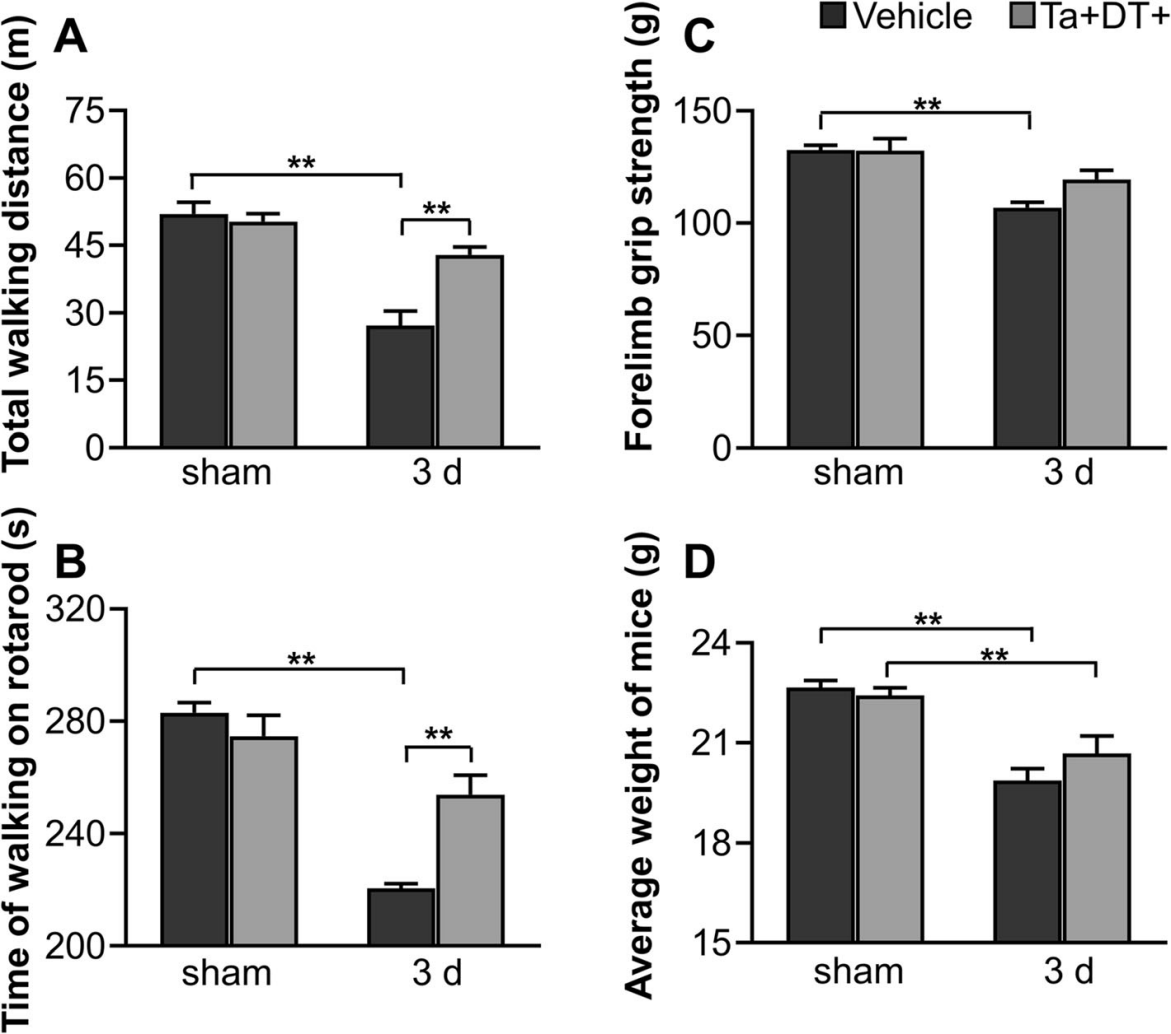
Effects of microglial depletion on mice behavioral performance and body weight. (**a**-**c**) Mice behavioral performance in spontaneous activity test, rotarod test, and grip strength test with or without ischemic stroke. (**d**) Body weight of mice with or without ischemic stroke. Microglial depletion did not show obvious effect on body weight of mice. Note that ischemia itself greatly impacted on behavioral performance and body weight (*n* ≥ 5, *p <0.05, **p <0.01)

Microglial activation is thought to be involved in functional recovery after ischemic stroke [34–36]. To study the effects of microglial depletion on behavioral performance, we compared the differences of body weight and motor ability between microglia-depleted mice and vehicle-treated mice. Consistent with results in other studies depleting microglia with small-molecule inhibitors [13, 37, 38], we did not observe significant motor dysfunction or weight loss in sham^Ta+DT+^ mice (Fig. 4), suggesting that microglia are not essential to these physiological functions. Three days after ischemic stroke, stroke^Ta−DT−^ mice showed much worse performance in the spontaneous activity test, grip strength test, and rotarod test than sham groups (Fig. 4a-c). Additionally, ischemic insult resulted in obvious loss of body weight (Fig. 4d). Statistical analysis showed a great improvement in motor ability after microglial depletion. Compared with stroke^Ta−DT−^ mice, the total distance traveled in spontaneous activity test of stroke^Ta+DT+^ mice increased by 57.47% and the time of walking on the rotarod by 15.16% 3 days post stroke, while the forelimb grip strength showed a slight rise (Fig. 4a-c). However, the mice presented no significant difference in body weight between stroke^Ta−DT−^ and stroke^Ta+DT+^ groups (Fig. 4d). These results indicated that depleting microglia was beneficial to behavioral recovery of mice at an early time point after stroke.

### Microglial depletion decreased inflammatory cells

**Fig. 5:**
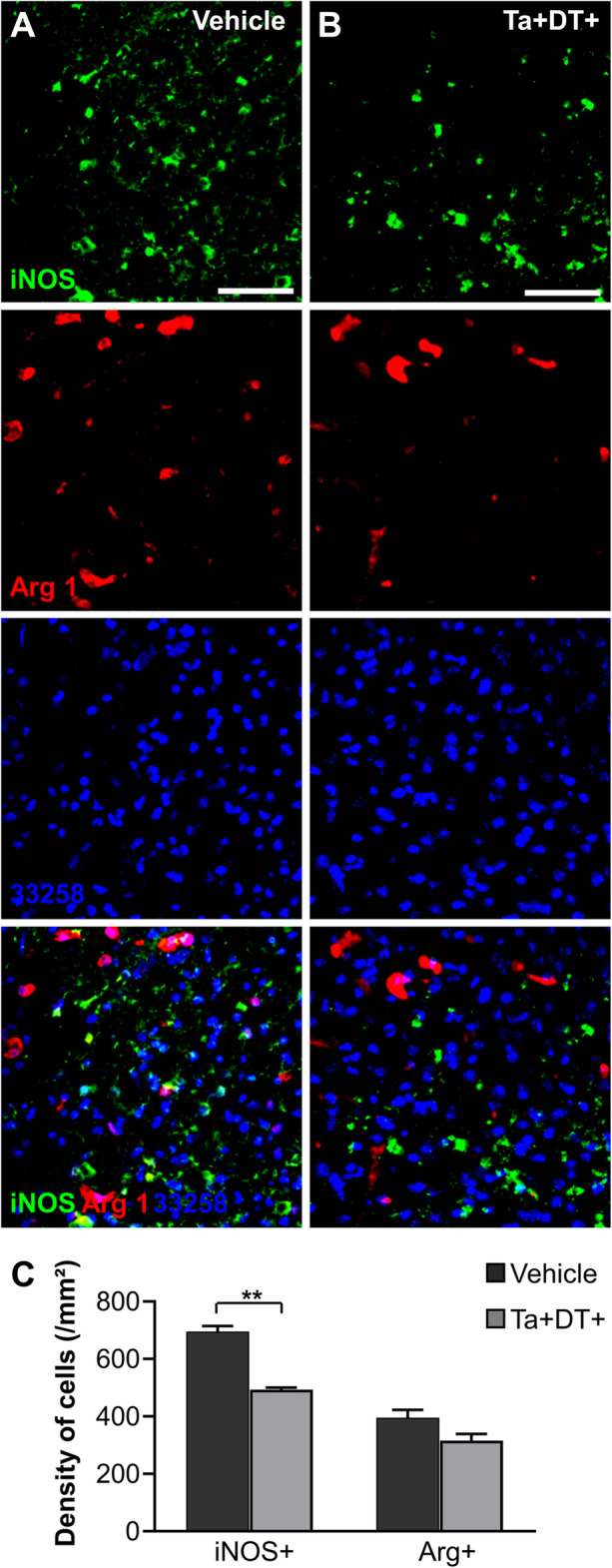
Depleting microglia reduced inflammatory cells at the lesion site. (**a**) Representative images showing iNOS^+^ cells and Arg1^+^ cells at the lesion site without microglial depletion. (**b**) Representative images showing iNOS^+^ cells and Arg1^+^ cells after microglia depletion (**a**-**b** Scale bars = 50 μm). (**c**) Densities of iNOS^+^ cells and Arg1^+^ cells in the periphery area of lesion site with microglia depletion or not (*n* = 4, *p <0.05, **p <0.01)

Inflammatory response is triggered quickly, lasting for several days after ischemic stroke [39]. To better understand the impact of microglial depletion on the ischemic damage, we quantitated the cells expressing typical inflammatory molecules iNOS and Arg1 after stroke. As expected, there was no iNOS^+^ or Arg1^+^ cells observed in the contralateral uninjured cortex (data not shown). We evaluated whether depleting microglia changed the densities of inflammatory cells at the lesion site. Our data showed that ischemia caused extensive expression of pro-inflammatory molecule iNOS and anti-inflammatory molecule Arg1 on brain cells 3 days after stroke (Fig. 5a, b). Cells expressing iNOS were found distributing throughout the ipsilateral ischemic area, while the Arg1^+^ cells were observed in the periphery of the ischemic area but not in the core zone (Additional file 1: Figure S1). The results showed that microglial depletion significantly decreased the density of iNOS^+^ cells by 29.13% (from 695.83 ± 19.16/mm^2^ in stroke^Ta−DT−^ mice to 493.13 ± 7.59/mm^2^ in stroke^Ta+DT+^ mice) (Fig. 5c). By contrast, the reduction of Arg1^+^ cells was less pronounced (20.26%, from 395.83 ± 27.28/mm^2^ in stroke^Ta−DT^ mice to 315.63 ± 23.46/mm^2^ in stroke^Ta+DT+^ mice) (Fig. 5c). These results indicated that microglial depletion in the early stage post stroke effectively reduced the detrimental inflammatory response in the ischemic region.

### Depletion of microglia altered the immune microenvironment in ischemic tissue

**Fig. 6:**
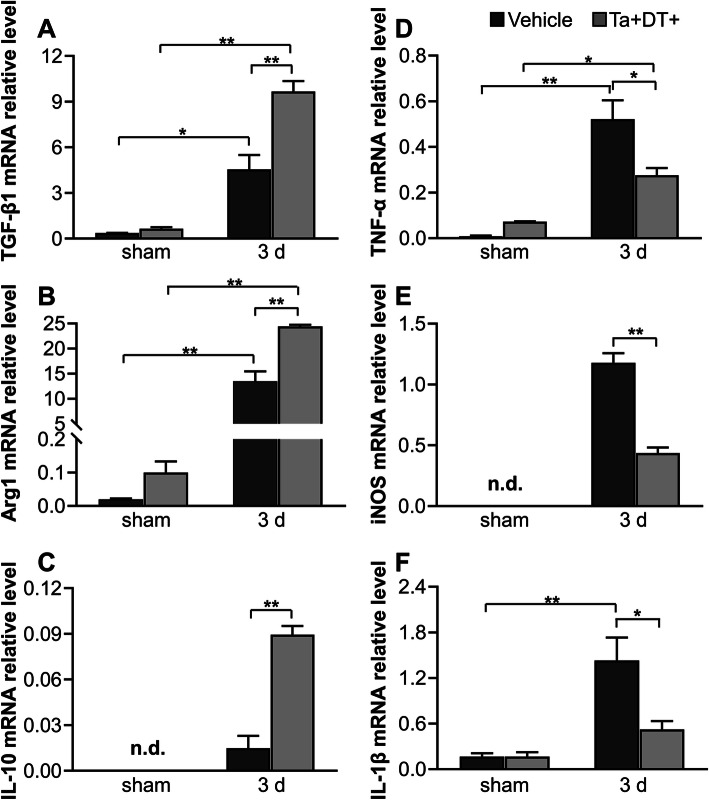
qRT-PCR analysis of mRNA expression of inflammatory factors in the presence and absence of microglia. (**a**-**c**) Relative mRNA levels of anti-inflammatory factors *TGF-β1*, *Arg1*, *IL-10* with and without microglial depletion. (**d**-**f**) Relative mRNA levels of pro-inflammatory factors *TNF-α*, *iNOS*, and *IL-1β* with and without microglial depletion (*n* ≥ 3, *p <0.05, **p < 0.01. n.d. = not detectable)

Microglia are a pivotal part of the inflammatory response in the brain [40]. To examine the effect of microglial depleting on immune microenvironment, we analyzed the relative mRNA expression of key immunomodulatory factors in stroke mice with or without microglial depletion. Using qRT-PCR, we first detected low mRNA expression of immunomodulatory factors in mice without ischemic stroke. There was no significant difference in mRNA level of *TGF-β1*, *Arg1*, *IL-10*, *IL-4*, *Ym1*, *iNOS*, and *IL-1β* between sham-^Ta−DT−^ and shame-^Ta+DT+^ mice. However, microglial depletion raised the expression of *TNF-α* and *MCP1* even without ischemia (Fig. 6, Additional file 2: Figure S2). At the 3rd day after ischemic insult, we detected an intense inflammatory response with all the tested factors upregulated (Fig. 6, Fig. S2). Intriguingly, microglial depletion had differential effects on the inflammatory factors. When resident microglia were depleted, we found that mRNA expression of anti-inflammatory factors increased (*Arg1* for 1.8-fold, *TGF-β1* for 2.1-fold, *IL-10* for 5.9-fold, *IL-4* for 4.5-fold, *Ym1* for 12.8-fold) (Fig. 6a-c, Fig. S2A, B), while pro-inflammatory factor decreased (*iNOS* for 0.4-fold, *IL-1β* for 0.4-fold, *MCP1* for 0.4-fold, *TNF-α* for 0.5-fold) 3 days post stroke (Fig. 6d-f, Figure S2C). These results indicated that depletion of microglia at an early stage after ischemic stroke diminished the pro-inflammatory response but facilitated the anti-inflammatory effects.

## Discussion

Previous studies revealed the rapid activation and expansion of microglia in the early phase of ischemia [7, 41, 42]. However, the role of microglia in ischemic stroke remains controversial. Many studies have shown that activated microglia have detrimental effects on ischemia outcomes, as inhibition of microglia activation with some drugs attenuates neurological deficit and the associated inflammatory response, and reduces blood-brain barrier (BBB) disruption and infarct size [14, 43–46]. Furthermore, the voltage-gated proton channel Hv 1 in activated microglia is responsible for ROS-mediated brain damage after ischemia [47, 48]. Nevertheless, there is also evidence suggesting that microglia activation is vital for reducing neuronal apoptosis, modulating inflammation [49–51], phagocytizing invading neutrophils and neuronal cell debris [52–54]. Moreover, transplantation of exogenous microglia enhances neuronal survival and neurogenesis after stroke [55, 56]. The dual roles of microglia are likely associated with different activation states under different models and drug treatments after ischemia. Experimental stroke models, such as tMCAo, pMACo, photothrombiotic stroke, and endothelin-1 induced ischemia, may have various pathological and cellular contexts which lead to different activation programs [57, 58]. Recently, microglia are thought to display distinct metabolic phenotypes exposed to different stimuli [59]. Drugs such as minocycline, adjudin, ticagrelor, and PNU282987 target different mechanisms related to microglia activation [45, 46, 50, 60], which could stimulate microglia differently and contribute to the dual functions of microglia. Although transplantation of exogenous microglia induces neuroprotection and behavioral improvement, immortalized microglial lines and primary cultured cells may behave differently from host microglia in the ischemic brain [55, 56].

In recent years, the field took advantage of agents to induce microglial ablation to investigate the effect of microglia on several brain pathologies. Many CSF1R kinase inhibitors are chosen for pharmacologic depletion of microglia with high effectiveness, such as PLX647, PLX3397, PLX5622, and Ki20227, with microglia rapidly repopulating after drug removal [13, 37, 61–64]. In addition, apoptosis inducer liposomal clodronate and immunotoxin Mac-saporin are used to specifically eliminate microglia [65–67]. The other approach is conditional genetic manipulation of microglia with suicide genes [68, 69] or to elicit their susceptibility to lethal drugs by the tamoxifen-inducible Cre recombinase system [25, 70]. Here, we investigated the effect of reactive microglia using a modified photothrombosis model combined with CX3CR1^CreER/+^:R26^iDTR/+^ mice and a selective microglial depletion system. In contrast to previous reports using CSF1R inhibitor and MCAo stroke model, which support neuroprotective effects of microglia in brain injury [13, 62], we found that selective ablation of microglia with CX3CR1^CreER/+^:R26^iDTR/+^ mice led to an evident reduction in infarct size and a better performance in motor ability after ischemic stroke, which reveal reactive microglia as accomplices to aggravate ischemic injury. The difference between previous studies and our current work may be due to different stroke models, depletion protocols, and depletion stages. It has been demonstrated that photothrombostic stroke lesion lacks normal penumbra and reperfusion [58], but possesses an accumulation zone of hypertrophic microglia [7]. Apoptotic neurons are detected earlier in a mouse photothrombotic stroke model than MCAo model [71]. These may result in higher activation of microglia and stronger inflammation in the photothrombotic stroke model than MCAo. In addition, microglial depletion method may have an impact on the experimental outcome. Evidence suggests that an increase in circulating neutrophils is detected as a result of Ta injections [72], which may effect the pathological progression. Given the efficacy of drugs, dosage dependent effect should be considered in depletion protocol. There are also concerns about the toxicity of Ta and DT treatment. High dose of TAM of Ta or DT may have significant toxicity and result in increased morbidity and mortality in mice [73, 74]. But low dosage of Ta and DT may affect the depletion efficiency [73]. It is important to choose the dosage and delivery method in the experimental procedure. Thus, the different impact on inflammatory cells following pharmacological or conditional genetic microglia depletion methods could influence the subsequent experiment. Furthermore, different depletion stage may also contribute to the controversial results. Activated microglia display different phenotypes and present distinctive spatial and temporal features after ischemia [42], and microglial depletion at acute or subacute ischemic stages may have different impact on stroke outcome. All of these aspects may lead to conflicting experimental results.

In line with the abatement of infarct volume upon microglia elimination, our histological results showed significant reduction in neurodegeneration and density of iNOS^+^ cells after stroke. Moreover, depletion of microglia had differential effects on inflammatory cytokines. On one hand, our data manifested significant downregulation of pro-inflammatory factors in the lesion site after microglial depletion which implied reactive microglia as the major source of pro-inflammatory molecules after stroke. Activated microglia produce a plethora of neurotoxic mediators such as NO, TNF-α, and IL-1β which have direct effects on neurologic outcomes of ischemic stroke [16]. Inhibition of iNOS, TNF-α, and IL-1β could attenuate ischemic injury and ameliorate neurological deficits [75–77]. On the other hand, we also found evident upregulation of anti-inflammatory mediators after microglial depletion. Although the increase of anti-inflammatory factors in the absence of microglia seems paradoxical, other brain cells likely serve as sources of inflammatory cytokines. Previous studies detected rapid production of neuronal IL-4 during sub-lethal ischemia [78] and upregulation of TGF-β1 and Arg1 in ischemic brain vessels [79, 80]. Under ischemic stress, reactive astrocyte also increases expression of neuroprotective IL-10 and Arg1 [81, 82]. Out of interaction with microglia, the altered cross-talk among other brain cells is likely to change and evoke their anti-inflammatory profiles after ischemic stroke. It is noteworthy that microglial depletion led to a significant increase in the mRNA expression of Arg1, but a slight decrease in the density of Arg1^+^ cells. This contradiction may be due to overexpression of *Arg1* mRNA concentrated in each Arg1^+^ cell, as well as post-transcriptional control and delay of protein translation of *Arg1* mRNA. Although our study supported a key role of microglial depletion in ischemic stroke, there are potential repercussions induced by microglial depletion. Evidence suggests that microglial depletion can cause a decrease in splenic macrophages and T cells and an increase in circulating neutrophils [72]. Further studies are warranted to assess the effect induced by the change of other cell types. Together, these data reveal that microglia play an essential role in the expression of immunomodulatory molecules at the ischemic site. Depletion of microglia skews the immune microenvironment of infarcted tissue toward an inflammation-suppressive state. Consequently, we propose that removing microglia or dampening microglial activation at an early stage of acute ischemic stroke may be helpful to limit neuronal injury and reduce cerebral ischemic damage.

## Conclusions

In summary, we proposed that specific depletion of reactive microglia effectively reduced pathological damage at the early stage of ischemic stroke, with smaller infarct volumes, reduced neurodegeneration, and improvement of motor activity in the absence of microglia. The results of immunohistochemistry and molecular biological assays confirmed that the immune microenvironment inclined to be inflammation-suppressive and neuroprotective to retard secondary damage to neurons and tissue in the lesion site when microglia were exhausted at this time. Further research is required to shed light on microglial functions at other periods and mechanisms of how reactive microglia impact inflammation and microenvironment in the brain after ischemia. Data from our behavioral evaluation additionally suggested that depletion of microglia in the early stage of ischemic stroke was conducive to behavioral recovery. These results present detrimental effects of activated microglia in the subacute phase of ischemia and offer a new target for early therapeutic strategy aimed at regulating the inflammation profile mediated by reactive microglia in lesion sites to reduce neuronal secondary damage and slow down the exacerbation of ischemic stroke.

## Supplementary Information

**Additional file 1: Figure S1.** Distribution of iNOS^+^ cells and Arg1^+^ cells in the lesion site 3 days after stroke. (A) Representative images of brain section stained with two typical inflammatory molecules in the presence of microglia. A mass of iNOS^+^ cells distributed all over the ischemic area, and a number of Arg1^+^ cells around the center. (B) Representative images showing a decline in number of iNOS^+^ cells and Arg1^+^ cells with microglial depletion (A-B Scale bar = 300 μm).

**Additional file 2: Figure S2.** mRNA expression of other inflammatory factors in the presence and absence of microglia. Relative mRNA levels of (A-B) anti-inflammatory factors *IL-4*, *Ym1* and (C) pro-inflammatory factors *MCP-1* with and without microglial depletion (n ≥ 3, *p <0.05, **p < 0.01. n.d. = not detectable).

## Data Availability

The data supporting the conclusions of this article are available from the corresponding author upon reasonable request.

## Abbreviations

Arg1: Arginase-1
BBB: Blood-brain barrier
CNS: Central nervous system
CSF1R: Colony-stimulating factor 1 receptor
DT: Diphtheria toxin
FACS: Fluorescence-activated cell sorting
FJC: Fluoro-Jade C
IL-1β: Interleukin 1-beta
IL-4: Interleukin 4
IL-10: Interleukin 10
iNOS: Inducible nitric oxide synthase
IRH: Infarcted area of the right hemisphere
MCP1: Monocyte chemoattractant protein-1
NO: Nitric oxide
NRH: Non-infarct region of the right hemisphere
PBS: Phosphate-buffered saline
pMACo: Permanent middle cerebral occlusion
qRT-PCR: Quantitative real-time polymerase chain reaction
ROS: Reactive oxygen species
Ta: Tamoxifen
TGF-β1: Transforming growth factor beta 1
TLH: Total left hemisphere
tMCAo: Transient middle cerebral occlusion
TNF-α: Tumor necrosis factor-α
YFP: Yellow fluorescent protein
Ym1: Chitinase 3-like 3
