# Supplementary figures and images for: Specific depletion of resident microglia in the early stage of stroke reduces cerebral ischemic damage
Source: J Neuroinflammation. 2021 Mar 23;18:81. doi: 10.1186/s12974-021-02127-w (PMC7986495; doi:10.1186/s12974-021-02127-w)

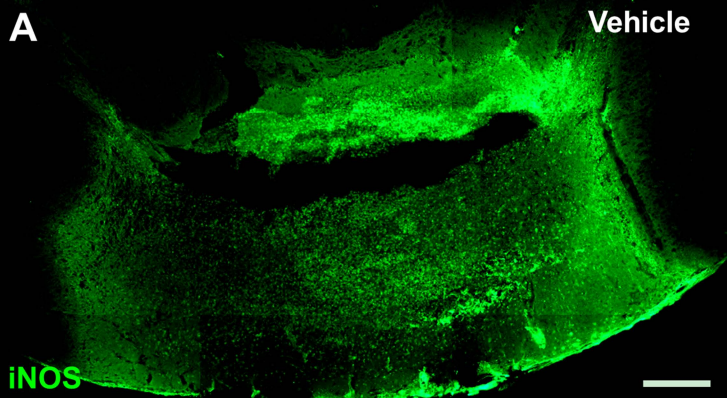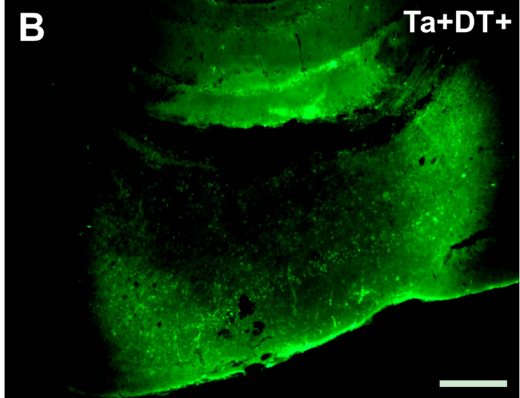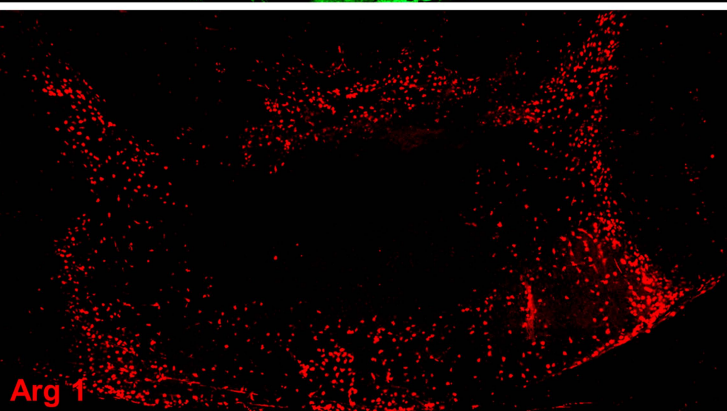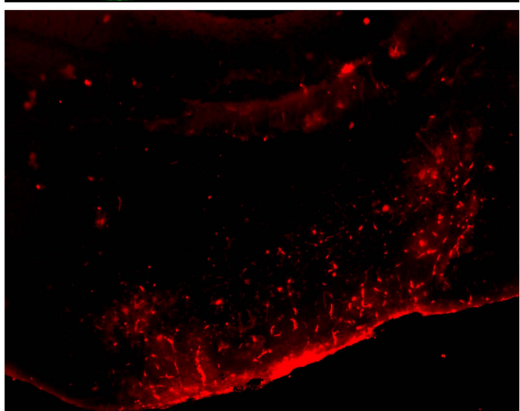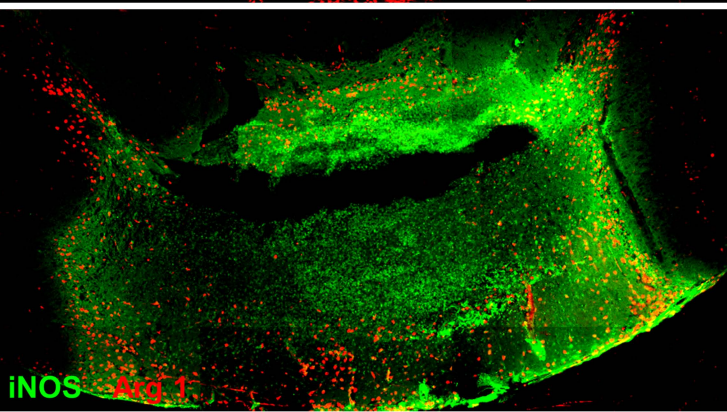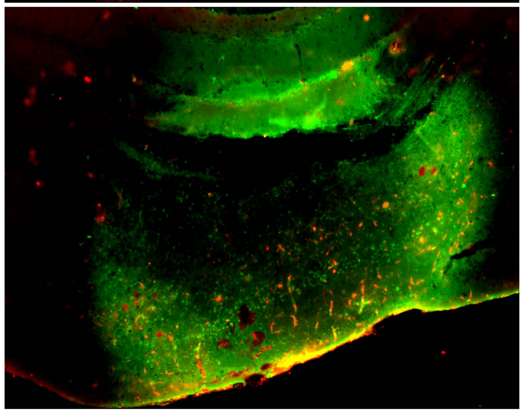

Supplement: Supplementary file 1 — Additional file 1: Figure S1. Distribution of iNOS+ cells and Arg1+ cells in the lesion site 3 days after stroke. (A) Representative images of brain section stained with two typical inflammatory molecules in the presence of microglia. A mass of iNOS+ cells distributed all over the ischemic area, and a number of Arg1+ cells around the center. (B) Representative images showing a decline in number of iNOS+ cells and Arg1+ cells with microglial depletion (A-B Scale bar = 300 μm). [file 12974_2021_2127_MOESM1_ESM.pdf]

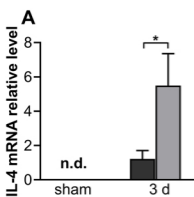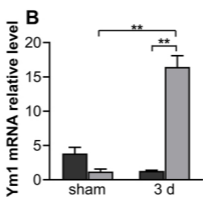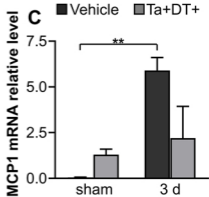

Supplement: Supplementary file 2 — Additional file 2: Figure S2. mRNA expression of other inflammatory factors in the presence and absence of microglia. Relative mRNA levels of (A-B) anti-inflammatory factors IL-4, Ym1 and (C) pro-inflammatory factors MCP-1 with and without microglial depletion (n ≥ 3, *p <0.05, **p < 0.01. n.d. = not detectable). [file 12974_2021_2127_MOESM2_ESM.pdf]
